# Structure and Technological Parameters’ Effect on MISFET-Based Hydrogen Sensors’ Characteristics

**DOI:** 10.3390/s23063273

**Published:** 2023-03-20

**Authors:** Boris Podlepetsky, Nikolay Samotaev, Maya Etrekova, Artur Litvinov

**Affiliations:** Micro- and Nanoelectronics Department, National Research Nuclear University MEPhI (Moscow Engineering Physics Institute), Kashirskoe Highway 31, 115409 Moscow, Russia

**Keywords:** MISFET, technological parameters, models, hydrogen sensor performances

## Abstract

The influence of structure and technological parameters (STPs) on the metrological characteristics of hydrogen sensors based on MISFETs has been investigated. Compact electrophysical and electrical models connecting the drain current, the voltage between the drain and the source and the voltage between the gate and the substrate with the technological parameters of the *n*-channel MISFET as a sensitive element of the hydrogen sensor are proposed in a general form. Unlike the majority of works, in which the hydrogen sensitivity of only the threshold voltage of the MISFET is investigated, the proposed models allow us to simulate the hydrogen sensitivity of gate voltages or drain currents in weak and strong inversion modes, taking into account changes in the MIS structure charges. A quantitative assessment of the effect of STPs on MISFET performances (conversion function, hydrogen sensitivity, gas concentration measurement errors, sensitivity threshold and operating range) is given for a MISFET with a Pd-Ta_2_O_5_-SiO_2_-Si structure. In the calculations, the parameters of the models obtained on the basis of the previous experimental results were used. It was shown how STPs and their technological variations, taking into account the electrical parameters, can affect the characteristics of MISFET-based hydrogen sensors. It is noted, in particular, that for MISFET with submicron two-layer gate insulators, the key influencing parameters are their type and thickness. Proposed approaches and compact refined models can be used to predict performances of MISFET-based gas analysis devices and micro-systems.

## 1. Introduction

Many types of hydrogen sensors are used in fire–explosion safety and environmental monitoring systems [1]. The microminiaturization and intellectualization of such systems based on microtechnology and nanotechnology as well as improving their performance characteristics are the main trends of their development [2]. To develop the integrated hydrogen sensors and gas-analytic lab-on-chip systems, the sensitive elements must have technological compatibility with the elements of the integrated circuits. The capacitor and transistor elements based on metal–insulator–semiconductor (MIS) structures have good compatibility with the integrated circuits’ elements [3].

Gas sensors based on MIS capacitors and field-effect transistors (MISFETs) have been studied by many investigators. A great contribution to the developments of gas-sensitive MIS devices has been made by the researchers at Linköping University since their first work in 1975 [4]. Works [5,6] described the gas sensitivity mechanisms, the kinetic modeling of hydrogen adsorption/absorption in thin films of catalytic metals and the formation of hydrogen atom dipoles in the metal–SiO_2_ interfaces of MIS sensors. MIS sensors with different gate material (palladium [6], platinum and iridium [7]), with dielectric films SiO_2_, Si_3_N_4_-SiO_2_, TiO_2_-SiO_2_ and Ta_2_O_5_-SiO_2_ have been investigated [8]. The semiconductors Si [4,7], GaAs [3,9] and SiC [10] were used in MIS gas sensors to detect the low concentrations of gases H_2_ [6], NH_3_ [7], H_2_S [11], NO_2_ [12] and CO [13]. The studies have shown that performance characteristics of MISFET-based hydrogen sensors depend on technological parameters [8], electrical modes [14], chip temperature [15] and external factors (e.g., irradiation [16]).

Work on the study of the hydrogen effect on the properties of thin Pd films (less than 1 micron) began long before the appearance of real MISFET, and they are still ongoing. It has been shown that under the influence of various concentrations of hydrogen in the air, the partial pressure of the surrounding gases, the temperature and type of substrate, the thickness and deposition technology of the Pd film as a result of chemical reactions, changes in the structure, density and chemical composition of palladium films can occur [8]. As a result, under certain conditions of thermo-hydrogen exposure, Pd_x_H_y_ and PdO compounds are formed in palladium films, and the films themselves can swell and peel off from the substrate [17]. These effects lead to irreversible and/or reversible changes in the electrical conductivity of the films and the work of the electron output from Pd [3], which is the physical principle of operation of some types of hydrogen sensors.

In addition to these effects, the following processes are considered in the models of hydrogen sensitivity of MIS capacitors and MISFET. Firstly, the hydrogen molecules adsorb on the surface of the Pd film and then dissociate into atoms. The hydrogen concentration in Pd is proportional to the concentration of adsorbed hydrogen molecules in Pd and hydrogen concentrations in air, as well as dependent on Pd temperature and the concentrations of other molecules [18]. Secondly, there is the diffusion of hydrogen atoms through the Pd film to the Pd–insulator interface [6,19]. Some hydrogen atoms form Pd_x_H_y_ compounds, the concentration, structure and “lifetime” of which strongly depend on the chip temperature and hydrogen concentration [14]. Some hydrogen atoms penetrate to the boundary, with the dielectric either directly through the pores in the palladium film or via the tunnel mechanism through palladium grains (clusters) [5,11]. It is these atoms that form a polarized dipole layer of H in the Pd–insulator interface [6]. In work [20], based on modeling, doubts are expressed about the possibility of forming a dipole layer at the palladium–dielectric boundary. Third, there may be diffusion and drift protons in the insulator [21].

Thus, the hydrogen sensitivity of MIS devices depends on many factors. The simultaneous taking into account of all the factors is very difficult or not possible at all. In this article, the hydrogen sensitivity of the sensor characteristics was evaluated on the basis of a two-component physical model that takes into account possible changes in the work of the electron output from the gate material and changes in the MIS structure.

In recent years, based on nano- on micro-technologies, gas sensors with low sensitivity thresholds and low inertia have been developed. For example, new developments used nanostructured palladium films and Pd nanotubes [22], electrodeposited nanomaterials [23], nanoporous silicon thin films [24], integrated FET [25] and carbon nanotubes [26]. Microtechnologies, in combination with CMOS technologies [27,28], are used not only for the development of hydrogen and hydrogen-containing gas sensors [29], but also for the detection of other types of gases (e.g., CO [13,30] and NO_2_ [12,31]). Note that sensor developments [13,26] are based on functionalized Single-Walled Carbon Nanotubes (SWNTs). 

The researchers at National Research Nuclear University MEPhI have developed and investigated the number of discrete and integrated gas sensors (MIS capacitors, Pd and Pt resistors) with Pd (or Pt)-SiO_2_-Si, Pd/Ti-SiO_2_-Si, Pd (or Pt)-Ta_2_O_5_-SiO_2_-Si structures. Experiments have demonstrated that MISFETs have the best performances compared to MIS capacitors and resistors. In addition, the integrated sensors, containing MISFET with a Pd(Ag)-Ta_2_O_5_-SiO_2_-Si-structure (hereinafter referred to as TSE), possess the best stability and reproducibility of characteristics [21]. In recent years, we have investigated the metrological and operating characteristics of TSE (e.g., electrical modes [14], chip temperature [15] and irradiation [16]).

In this paper, we have investigated the influence of the structure and technological parameters (STPs) on the characteristics of MISFET hydrogen sensors in a general form, and for TSE, based on refined compact models, allowing us to simulate the hydrogen sensitivity of gate voltages or drain currents in weak and strong inversion modes, taking into account changes in the work of the electron output from the gate material and charges in the MIS structure. A quantitative assessment of the effect of STP on the TSE conversion function, hydrogen sensitivity, gas concentration measurement errors, sensitivity threshold and operating range is given.

## 2. Materials and Methods

### 2.1. Initial Structure and Technological Parameters of TSE

The initial structure and technological parameters, which are considered unchanged when modeling sensor characteristics, included the dimensional parameters of the structural elements, the parameters of the semiconductor and the dielectric materials listed in Table 1. The STP values are determined using the specified materials, structure and topology of the TSE and are used to calculate the parameters *C*_0_, *a* and *b* of the TSE characteristic electrophysical models.

The errors in the calculations of electrophysical parameters depend on the errors of the STPs, which are determined using the technological standards or errors of the measuring instruments, if the values of the STPs were determined experimentally. For example, if ε_3_ is (12 ± 0.2), *w* is (3.2 ± 0.02) mm, *L* is (10 ± 0.1) μm, µ*_n_* is (200 ± 5) cm^2^/(V∙s), *N_A_* is (5 ± 0.02) × 10^15^ cm^−3^, then the relative errors of the parameters *C*_0_, *a* and *b* are equal to 3.5%, 4.5% and 8.7%, respectively. 

Denote the parameters with the symbol *p_k_* ∈ {*p_k_*} (*k* = 1, 2, …, 13 in Table 2). In general, the absolute and relative errors of the parameters Δ*p_k_* and δ*p_k_* are equal to ׀*p_kn_* − *p_k_*׀ and (Δ*p_k_*/*p_kn_*) × 100%, respectively. The value of *p_kn_* is the desired (nominal) value of parameter *p_k_* or its average value, if this parameter was determined experimentally from a set of measured values.

The absolute errors of Δ*p_k_* depend on the film manufacturing technologies (for parameters with indices *k* ∈ {1; 2; 3; 4; 5; 6}, on photolithography technologies (for *p*_8_, *p*_9_), on methods of estimating their thicknesses (for parameters with indices *k* ∈ {4; 5; 6} and on the type and structure of the materials (for parameters with indexes *k* ∈ {11; 12; 13}. Typically, the values of Δ*w* and Δ*L* are in the range of 0.1 to 0.5 microns, and values Δ*d* ∈ [5 nm; 15 nm] depend on *d*. The relative errors δ*w* and δ*L* are in the range from 0.002%...0.01% to 1.1%...5%. For specific technologies of the semiconductor wafers and dielectric films production, the relative errors δε_1_, δε_2_, δε_3_, δ*µ_n_* and δ*N*_A_ usually do not exceed 2%. The relative errors of δ*d*_1_ and δ*d*_2_ are in the range of δ*d*_min_ ∈ [2%; 10%] to *d_max_* ∈ [7.5%; 30%] and are the maximum for the STPs. Consequently, the thicknesses *d*_1_ and *d*_2_, the dimension of which will be determined in nm, can be considered the most critical STP.

### 2.2. Metrological Characteristics of TSE

To assess the influence of various factors on the characteristics of the sensors and systems being developed based on MISFETs, simulation modeling can be used, which is considered a fast and inexpensive method compared to full-scale tests of sensors with various STPs. In simulation modeling, the type, structure and values of STPs and the parameters of the models are chosen arbitrarily. In this paper, the characteristics of the TSE with the parameters specified in Table 1 were studied.

**Figure 1:**
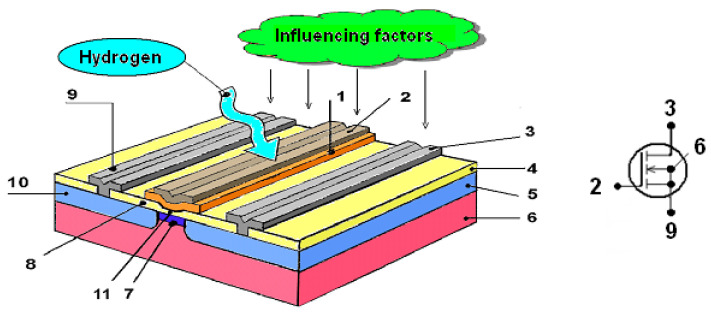
The structure and the schematic designation of TSE: 1 is gas-sensitive film, 2 is metal gate, 3 and 9 are drain and source contacts, 4 and 8 are passivating films, 5 and 10 are drain and source, 6 is substrate, 7 is channel, 11 is thin gate dielectric.

**Figure 2:**
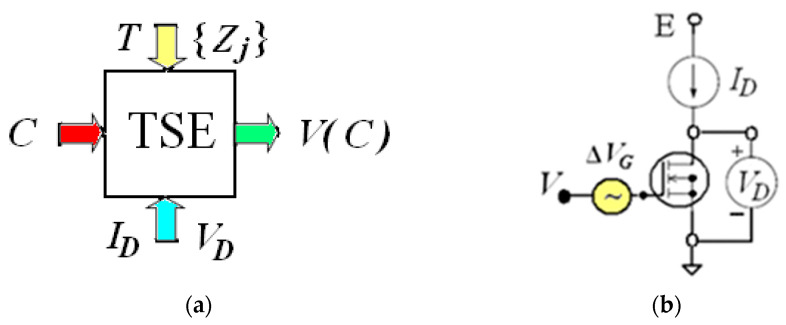
(**a**) The input informative parameter is the gas concentration *C*; the output informative parameter is the voltage of the output signal *V*; the operating chip temperature is *T*; the parameters of the electrical mode of the circuit are the current *I_D_* and the voltage *V_D_*; external influencing factors {*Z_j_*} are molecules of other gases, temperature, humidity and radiation background of the environment. (**b**) The circuit for measuring the dependence *V* as function of *C*.

A fragment of the structure, the schematic designation of the *n*-channel TSE and a potentiometric circuit of its embedding for measuring the gas concentration *C* are shown in Figure 1 and Figure 2.

The main metrological characteristics of TCE include: conversion function (dependence *V* as function of *C*); differential *S_d_* and integral *S* sensitivities being equal to d*V*/d*C* and Δ*V*/Δ*C*; absolute ΔC and relative δ*C* errors of measurement *C*; the sensitivity threshold *C_th_*; minimum relative error δ*C_min_*; the boundaries of the working range *C*_1_ and *C*_2_, limited by a given error δ*C_max_* and finally *C_max_*.

As previous studies [16,21] have shown, the conversion function of a TSE can be represented in general form as *V* (*C*) is [*V*_0_ − Δ*V*(*C*)], where the initial value of *V* (at *C* = 0) is equal to *V*_0_. In general, the output signals *V* of MISFETs-based sensors are formed as a result of the embedding of one or more transistors in various measurement circuits. In this paper, the main components of the models are determined using experimental studies of one TSE in the mode with a constant current *I_D_* and the voltage *V_D_* as shown in Figure 2b. In this case,

*V* (*C*) = *V_G_* (*C*) = *V_G_*_0_ − Δ*V_G_* (*C*) and *S_d_ =* d*V_G_/*d*C,* (1)

*V_G_*_0_ (φ*_s_*) = φ*_s_* + *a*·{φ*_s_* + φ*_T_*·exp[(φ*_s_* − 2φ*_s_*_0_)/φ*_T_*)]}^1/2^ + φ*_ms_*_0_ − [*Q_te_*_0_ + *Q_ss_* (φ*_s_*)]/*C*_0_. (2)

For modeling the characteristics of TSE sensors, the following expression can be used to approximate the conversion function in the range of hydrogen concentrations from 0.005 vol.% to 1.5 vol.% [16]:

Δ*V_G_*(*C*) = Δ*Q_te_*(*C*)/*C*_0_ − Δφ*_ms_*(*C*) = Δ*V_m_* · [1 − exp(−*k_C_*·*C*)] > 0. (3)

Then, the sensitivity of *S_d_* is equal to [−*k_C_*·Δ*V_m_* · exp(−*k_C_*·*C*)]. The maximum relative error of measuring the gas concentration δ*C* in the general case is represented as

δ*C* = 100% × Δ*C*/*C* = 100% × [Δ(*C*)·**|***S_d_***|** + Δ*V* + Δ(*V*)]*/*(*C***|***S_d_***|**), (4)

where Δ(*C*) is the absolute error of the specified gas concentration during sensor calibration, Δ*V* is the error voltage measurements and Δ(*V*) being equal to Δ(Δ*V_G_*) is the absolute voltage error associated with an absolute error of the parameter *p_k_* and is approximately equal to [d*V_G_/*d*p_k_*]·Δ*p_k_*. Then, after sensor calibration the sensitivity threshold *C_th_* is (Δ*V/***|***S_dmax_***|**), minimum relative error is δ*C_min_* and the boundaries of the working range *C*_1_ and *C*_2_ can be determined as solutions of Equations (5) and (6). The value of δ*C_min_* is δ*C* (at *C* is C^*^), where C^*^ is the solution of the following equation:

d(δ*C*)/d*C* = 0. (5)

The values of *C*_1_ and *C*_2_ are solutions of Equation (6) with respect to *C* at a given δ*C_max_* greater than δ*C_min_*:

δ*C_max_* = 100% × [Δ(*C*)**|***S_d_***|** + Δ*V* + Δ(*V*)]*/*(*C***|***S_d_***|**)*,* (6)

*C_max_* = (1/*k_C_*) · ln (Δ*V_m_*/Δ*V*). (7)

These transcendental equations will be solved using numerical methods. The testing *n*-channel MISFET based on the Pd-Ta_2_O_5_-SiO_2_-Si structure was fabricated on a single chip (2 × 2 mm^2^) together with a (*p–n*) junction temperature sensor and heater resistor by means of conventional *n-*MOS technology. Technological processes are detailed and presented in [14,21].

## 3. Results

### 3.1. Influence of Film Manufacturing Technology and Thicknesses on the Components of the Conversion Function

The experimental studies demonstrated that the MISFET hydrogen sensitivity depends on several effects, which occur in regions of the (ambient gas)–metal–dielectric structure. The probability of various effects depends on the following factors: chip temperature; partial pressure of hydrogen; material, chemical composition, structure, manufacturing technology and thickness of the gate film and the pressure of the gas medium [4,13,18,22,23]. The presence of a large number of influencing factors complicates and/or make it impossible in principle to simulate the influence of the material, the technology of production and the thickness of the shutter film on the metrological characteristics of MISFETs. The degree of this influence can only be determined experimentally. The morphology (crystalline, polycrystalline, amorphous, nanoparticles, etc.) of palladium films may affect the hydrogen sensitivity of sensors based on Pd film resistors or MIS structures as shown, for example, in [21,22].

Basically, the deposition technology and thickness of metal film *d_m_* can affect the conversion function components Δ*Q_te_*(*C*) and Δφ*_ms_*(*C*). In the investigated TSE Pd film (*d_m_* is 70 ± 5 nm) was prepared using laser evaporation in vacuum 2·10^−3^ Pa in the substrate’s temperature range of 300–400 °C. For this technology, at thicknesses *d_m_* that is less than 80 nm, the palladium film has a porous structure. Therefore, it can be assumed that the hydrogen sensitivity and response time will be independent of *d_m_* and of its deviations Δ*d_m_* from the nominal values of *d_mn_*. For other technologies (for example, with thicknesses of nanostructured Pd films less than 30 nm), the response time decreased, and the sensitivity can be increased at low concentrations (~5–50 ppm) with a decrease in *d_m_* [32]. Quantitative analysis of the effect of the Pd films’ characteristics on the hydrogen sensitivity of the TSE requires special experimental studies, which have not been observed in this work.

Deposition technologies and the thicknesses of dielectric films *d*_1_ and *d*_2_ can affect the conversion function components *V_G_*_0_ and Δ*V_m_*, which according to (2) and (3) are inversely proportional to specific capacity *C*_0_:

*V_G_*_0_ = φ*_s_* + 0.085 + {41·[φ*_s_* + 0.033·exp((φ*_s_* − 0.42)/0.033)]^1/2^ − 5}/*C*_0_ (V), (8)

Δ*V_m_* = Δ*Q_tem_*/*C*_0_ − Δφ*_msm_* = 15/*C*_0_ + 0.11 (V), (9)

*C*_0_ = (ε_0_ε_1_ε_2_)/(ε_1_*d*_2_ + ε_2_*d*_1_), (10)

where in engineering physical models (8) and (9), the dimensions of the potentials are the volts, charge density is nCl/cm^2^, capacitance is nF/cm^2^ and thicknesses is nm. With an increase in the hydrogen concentration, the work of the electron output from Pd decreases, and therefore you have a negative value Δφ*_ms_*(*C*) [3]. The absolute Δ*C*_0_ and relative δ*C*_0_ being equal to 100% × Δ*C*_0_/Δ*C*_0_ errors for *C*_0_ are, respectively, equal to:

Δ*C*_0_ = ε_0_[ε_2_*d*_1_(Δε_1_·ε_2_ + 2Δε_2_·ε_1_) + ε_1_*d*_2_(Δε_2_·ε_1_ + 2Δε_1_·ε_2_) + ε_1_·ε_2_(Δ*d*_2_·ε_1_ + Δ*d*_1_·ε_2_)]/(ε_1_*d*_2_ + ε_2_*d*_1_)^2^, (11)

**Figure 3:**
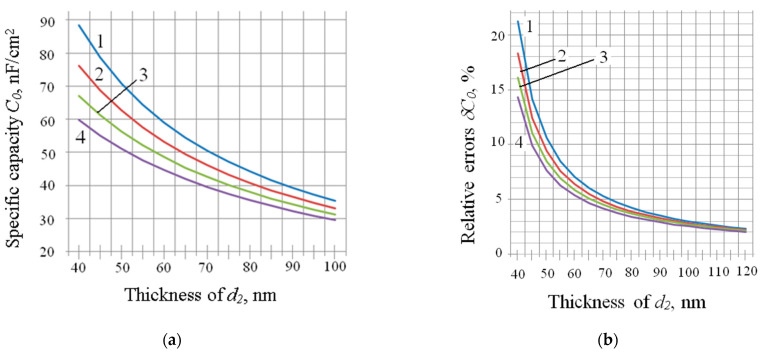
(**a**,**b**) are dependences *C_0_* (*d_2_*) and *δC_0_*(*d_2_*) at *d_1_*: 1 → 0 nm; 2 → 40 nm; 3 → 80 nm; 4 →120 nm.

δ*C*_0_ = [δε_1_(2ε_1_*d*_2_ + ε_2_*d*_1_) + δε_2_(2ε_2_*d*_1_ + ε_1_*d*_2_) + δ*d*_2_·ε_1_*d*_2_ + δ*d*_1_·ε_2_*d*_1_]/(ε_1_*d*_2_ + ε_2_*d*_1_), (12)

where Δε_1_, Δε_2_, Δ*d*_1_ and Δ*d*_2_ and δε_1_, δε_2_, δ*d*_1_ and δ*d*_2_ are the absolute and relative errors of the corresponding values. For the values, ε_1_ is 25 and ε_2_ is 4, and the dependences of the components *C*_0_ and δ*C*_0_ on thicknesses of *d*_2_ at different values of *d*_1_ are shown in Figure 3.

**Figure 4:**
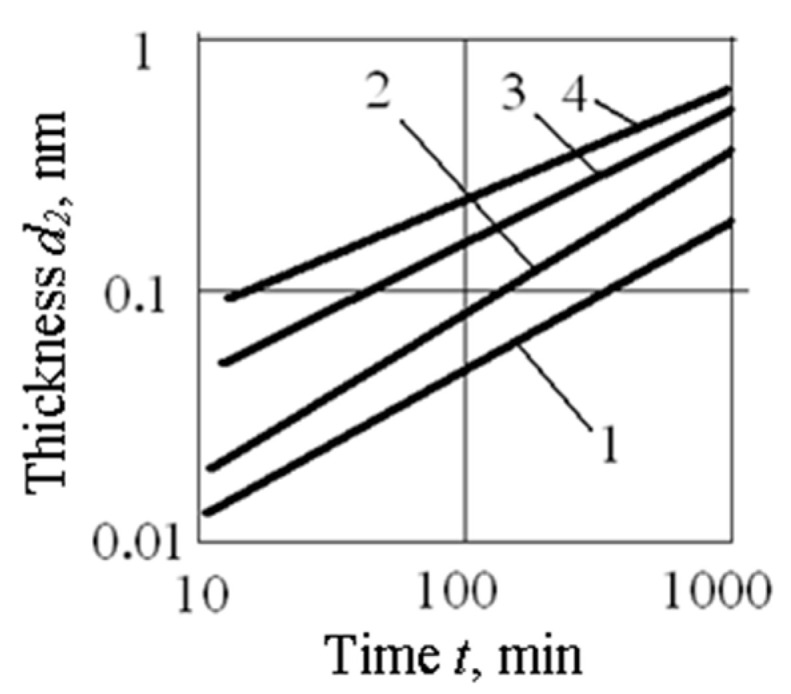
Dependence of the SiO_2_ film thickness *d*_2_ on the oxidation time *t* in dry oxygen at temperatures: 1 → 900 °C; 2 → 1000 °C; 3 → 1100 °C; 4 → 1200 °C.

In the process of forming metal and dielectric films at a constant temperature *Tp* of the silicon wafer (substrate), their thicknesses depend on the time of the process *t* and on the temperature-dependent growth rate of the film *v_i_*. For example, when creating a SiO_2_ film via silicon oxidation in dry oxygen, the dependence of *d*_2_(*t*) for various *Tp* is shown in Figure 4 (based on Figure 8a in [32]). At the initial stage of silicon oxidation (up to *d*_2_ is about 30 nm), the thickness of the SiO_2_ film is proportional to the oxidation time *t*; with further oxidation, the thickness of *d*_2_ is proportional to (*t*)^1/2^. If the oxidation of silicon in dry oxygen is carried out at a temperature of 1100 °C, then the thickness *d*_2_ is *v*_1_·*t* (nm) at *t* ∈ [0; 10 min] and *d_2_* = 30 + *v*_2_·(*t*)^1/2^ (nm) when the *t* is greater than 10 min, where *v*_1_ and *v*_2_ are equal to 4.0 nm/min and 8.5 nm·min ^½^; [*t*] is min.

The error δ*d*_2_ is (δ*v*_2_ + 100% × Δ*t*/*t*) or δ*d*_2_ is (δ*v*_2_ + 50% × Δ*t*/(*t*)^1/2^), where δ*v*_2_ depends on the dispersion of the plate temperature Δ*Tp*. The value of Δ*t* is determined by the time parameters of the temperature response *Tp* (*t*) when the plate is introduced into the core with the constant temperature and partial pressure of oxygen, or by the time parameters of establishing a constant partial pressure of oxygen after oxygen is introduced into the placement zone of the plate heated to the operating temperature *Tp* and the time of oxygen pressure drop. Usually, δ*v*_2_ < 0.5% and Δ*t* ∈ (0.5; 2) min. For a given thickness *d*_2_, the error δ*d*_2_ can be estimated as δ*d*_2_ being less than the value of [0.5% + 425Δ*t*/(*d*_2_ − 30)]. For example, if *d*_2_ is 50 nm and Δ*t* is 30 s, then the value of δ*d*_2_ < 11.1%, and if *d*_2_ is 80 nm and Δ*t* is 30 s, then the value of δ*d*_2_ < 4.75%.

Thus, the thicknesses and dielectric permittivities of dielectric films determine the capacitance *C*_0_, which can be considered a key parameter affecting the TSE conversion function.

### 3.2. Influence of Material’s Parameters and Topological Dimensions of Elements on Conversion Function Components

The material’s parameters (ε_3_, *N*_A_, *µ_n_*) and topological dimensions (*L*, *w*) affect parameters *a* and *b*, which depend on the value of *C*_0_. According to (2), the conversion function component *V_G_*_0_ depends on the variables parameters *a* and φ*_s_*. Then, the absolute error Δ*V_G_*_0_ with small deviations Δ*a* and Δφ*_s_* a from their average values can be presented as:

Δ*V_G_*_0_ = (∂*V_G_*_0_*/*∂φ*_s_*)Δφ*_s_* + (∂*V_G_*_0 */*_∂*a*)Δ*a* = 0.01·(*K*_φ_·φ*_s_*·δφ*_s_* + *K_a_*·*a*·δ*a*), (13)

*K*_φ_ = 1+ 0.5*a*(1+ exp *m*)/(φ*_s_* + φ*_T_*·exp *m*)^1/2^; *K_a_* = (φ*_s_* + φ*_T_*·exp *m*)^1/2^; *m* = (φ*_s_* − 2φ*_s_*_0_)/φ*_T_*. (14)

Parameter *a* depends on values of ε_3_, *N*_A_ and *C*_0_, and the potential φ*_s_* depends on the given values of *I_D_* and *V_D_*. According to the simplified TSE electrical model [15], the dependence *I_D_* (*V_G_*) at φ*_s_* > 2φ*_s_*_0_ has two sections: parabolic when *I_D_* ∈ [*I_D_*_0_; *I_D_*_1_] and φ*_s_* ∈ [2φ*_s_*_0_; φ*_s_*_1_], and linear when value of *I_D_* is greater than *I_D_*_1_ being equal to (*I_D_*_0_ + 0.5*bV_D_*^2^). In principle, the entire range of changes in the drain current *I_D_* and gate voltage *V_G_* corresponding to the inversion mode can be used to measure the hydrogen concentration. Usually, the values of the set current are within the error range: *I_D_* is *I_Dn_* ± Δ*I_D_*.

In practice, the electric mode of strong inversion (at φ*_s_* > φ*_s_*_1_) is chosen, in which the measurement errors Δ*V_G_*_0_ and δ*C* are minimal. For example, when *V_D_* is 0.2 V (*I_D_*_1_ is 42 μA) and *I_D_* is equal to (20 ± 2) μA, the value of *V_G_*_0_ is (1.16 ± 0.01) V, and when *I_D_* is equal to (100 ± 2) μA, the value of *V_G_*_0_ is (1.25 ± 0.005) V. Then, the values of *I_D_* and fluctuations of Δφ*_s_* are represented as:

*I_D_* = *b*·{*a*·*V_D_*·[(φ_s_ + φ*_T_* exp *m*) ^1/2^ − φ*_s_*^1/2^] − 0.5*V_D_*^2^}; Δφ_s_ ≈ 2Δ*I_D_*·(φ_s_ + φ*_T_* exp *m*)^1/2^/[*a*·*b*·*V_D_*·(1 + exp *m*)]. (15)

**Figure 5:**
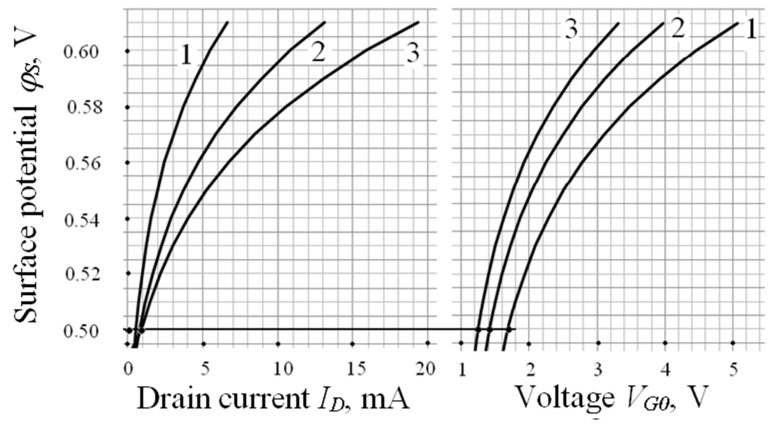
Dependences of values φ*_s_* on the current *I_D_* at different *V_D_* (1→ 0.1 V; 2 → 0.2 V; 3 → 0.3 V), and on values of *V_G_*_0_ at different *C_0_* (1→ 30 nF/cm^2^; 2 → 40 nF/cm^2^0.2 V; 3 → 50 nF/cm^2^).

The dependences of the potential φ*_s_* on the current *I_D_* at different *V_D_* and on values of *V_G_*_0_ at different *C*_0_ are shown in Figure 5. The relative errors of conversion function components *a* and *b* are equal to:

δ*a* = δ*C*_0_ + 0.5(δε_3_ + δ*N_A_*) = 4.5% and δ*b* = δ*C*_0_ + δ*µ_n_* + δ*w* + *δL* = 8.7%. (16)

Quantitative values of *a* and *b* are given for the investigated TSE. Average values of the parameters of the conversion function for different *C_0_* and (*w*/*L*) are presented in Table 3. As an example, Figure 5 shows the coordinates of the points corresponding to the values *V_G_*_0_ being equal to 1.2 V, 1.4 V and 1.7 V for different *V_D_* and a given drain current of 1 mA.

### 3.3. Influence of Specific Capacity on the Main Metrological Characteristics of TSE

**Figure 6:**
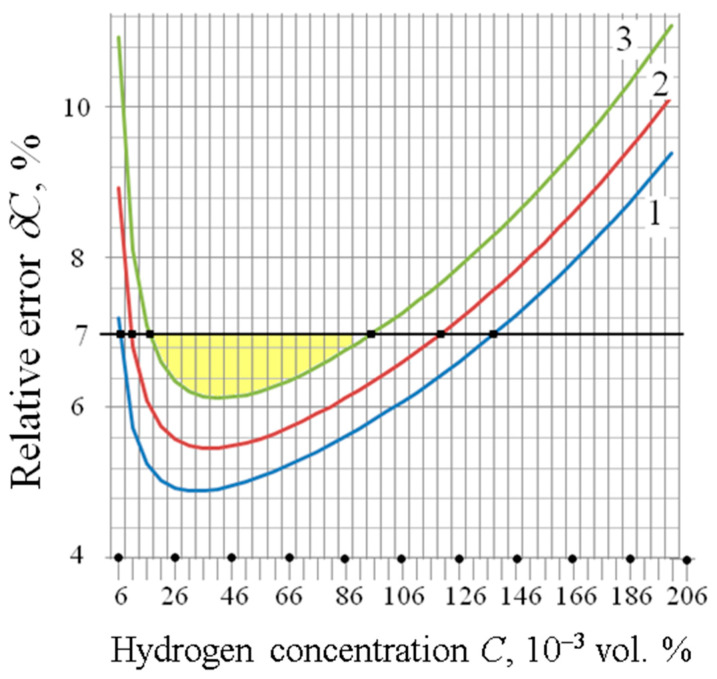
Dependence of δ*C*(*C*) for different *C*_0_: 1 → 30 nF/cm^2^; 2 → 40 nF/cm^2^; 3 → 50 nF/cm^2^. The values of *C*_1_ and *C*_2_ correspond to the square points on the curves. The color indicates the working area for the curve corresponding to the capacity of 50 nF/cm^2^.

The quantitative assessment of the effect of STPs on the initial value of the output signal, hydrogen sensitivity, absolute and relative errors in measuring gas concentration, the sensitivity threshold, the maximum concentration and the hydrogen concentration range for a given maximum relative error is given. The calculations used engineering physical models obtained on the basis of selected electrophysical and electrical models. The dependences of *δC*(*C*) and the average values of metrological characteristics for different *C_0_* are presented in Figure 6 and in Table 4.

## 4. Discussion

The analysis of the data obtained allows us to draw the following conclusions.

- All the considered STPs of the TSE (*p_k_* in Table 2) affect the components of the conversion function on which the main metrological characteristics depend.
- The deposition technology and thickness of the metal film dm can affect the conversion function components ΔQ*_te_*(C) and Δφ*_ms_*(C) on which the sensor’s hydrogen sensitivity and the response time depend. A quantitative analysis of the effect of the Pd films technological characteristics on TSE hydrogen sensitivity requires special experimental studies. In the investigated TSE, the Pd film has a porous structure. Therefore, it can be assumed that the hydrogen sensitivity and response time will be independent of d*_m_* and of its deviations Δd*_m_* from the nominal values of d*_mn_*.
- The values of *V_G_*_0_ and Δ*V_G_*_0_ depend on *N_A_*, *C*_0_ and the given electrical parameters *I_D_* and *V_D_*. With the growth of *I_D_* and *V_D_*, the error Δ*V_G_*_0_ decreases. As a result of the calibration of the measuring device, the zero error is determined by the error Δ*V_G_*_0_ or the instrumental error of the voltage measurement ΔV (in the examples considered, ΔV is 1 mV).

## 5. Conclusions

Refined compact electrophysical and electrical models are proposed that link the drain current, the voltage between the drain and the source, the voltage between the gate and the substrate with the structure and technological parameters (STPs) of an *n*-channel MISFET as a sensitive element of a hydrogen sensor (TSE). The method of the analytical assessment of the effect of STPs on the main metrological characteristics of a TSE is proposed. A qualitative assessment is given of the influence of the metal gate film manufacturing technology, the technology of manufacturing the gate dielectric films and their thicknesses, the concentration of impurities in the semiconductor, the length and width of the channel and the mobility of electrons in the channel on the metrological characteristics of TCE.

Using the example of a TSE with a Pd-Ta_2_O_5_-SiO_2_-Si structure, manufactured according to a specific technology, a quantitative assessment of the effect of STPs on the initial value of the output signal, hydrogen sensitivity, absolute and relative errors in measuring gas concentration, the sensitivity threshold and the hydrogen concentration range for a given maximum relative error is given. The calculations used engineering physical models obtained on the basis of selected electrophysical and electrical models.

The degree of influence of each STP and their errors on the components of the conversion function and the main metrological characteristics of a TSE is shown. It has been established that for a specific technology of manufacturing TSEs (in particular, for MISFET with submicron two-layer gate insulators), the key influencing parameters are their type and thickness. Proposed approaches and models can be used to predict performances of MISFET-based gas analysis devices and micro-systems and also to solve the inverse problem determining the STPs according to the specified metrological characteristics.

In contrast to works [22,29], in which structures with ultrathin single-layer dielectric films were studied, this work shows the significant effect on the hydrogen sensitivity of sensors of submicron thicknesses of MISFET dielectrics.

## Figures and Tables

**Table 1:** Average values of the parameters of MISFETs and parameters of used models.

Symbols	Parameters	Values
Parameters of semiconductor and dielectric materials		
ε_1_, ε_2_ and ε_3_	relative permittivity of Ta_2_O_5_, SiO_2_ and Si	25, 4 and 12
*N*_A_	concentration of acceptors in Si	5 × 10^15^ cm^−3^
*µ_n_*	electron mobility in the channel	200 cm^2^/(V∙s)
Dimensions of structural elements		
*L* and w	channel length and width	10 μm and 3.2 mm
*d*_1_ and *d*_2_	thicknesses of Ta_2_O_5_ and SiO_2_	90 nm and 80 nm
*d_m_*	thickness of the gate metal film	70 nm
Constants and derived parameters		
ε_0_	dielectric constant of vacuum	8.85 × 10^−12^ F/m
*k*	Boltzmann constant	1.38 × 10^−23^ J/K
*q*	electron charge	1.6 × 10^−19^ Cl
*d*	thickness of the gate dielectric is (*d*_1_ + *d*_2_)	170 nm
ε	effective permittivity of the dielectric layer is (*d*ε_1_ε_2_)/(ε_1_*d*_2_ + ε_2_*d*_1_)	7.1
*C*_0_	specific capacity of the dielectric (ε_0_ε)/*d*	37 nF/cm^2^
*a*	charge parameter in Si is (2*q* ε_0_∙ε_3_∙*N_A_*)^1/2^*/C*_0_	1.18 V^1/2^
*b*	specific steepness is *(*µ*_n_w C*_0_*)/L*	2 mA/V^2^
Physical and electrical parameters		
φ*_ms_*	output work difference potential Pd– Si	φ*_ms_*_0_ = 85 mV
*T*	chip temperature	400 K
φ*_T_*	thermal potential (*kT/q*) at 400 K	33 mV
φ*_gb_*	the potential of the band gap in Si	1.08 V
φ*_s_*_0_	the potential of acceptors’ level is φ*_T_* ln(*N_A_/n_i_*) at 400 K	0.21 V
φ*_s_*	surface potential is [φ(SiO_2_ − Si) − φ*_F_*]	0.2…0.8 V
*Q_te_* and *Q_ss_*	charge densities in the dielectric and in SiO_2_ − Si interface	(5…100) nKl/cm^2^
*I_D_*	drain current	(2…300) μA
*V_D_*	voltage between the drain and the source	(0.1…0.5) V
*V_G_*	voltage between the gate and the substrate	(1…3) V
*Q_te_* and *Q_ss_*	charge densities in the dielectric and in SiO_2_ − Si interface	(5…100) nCl/cm^2^
*I_D_*	drain current	(2…300) μA
*V_D_*	voltage between the drain and the source	(0.1…0.5) V
*V_G_*	voltage between the gate and the substrate	(1…3) V

**Table 2:** The effect of STP *p_k_* on the components of TSE models.

k	STP		Components of TSE Models
1	film production technologies	Pd	ΔQ_te_ (C); Δφ_ms_(C); φ_ms0_
2		Ta_2_O_5_	ΔQ_te_(C); C_0_; b
3		SiO_2_	Q_te0_; Q_ss_; C_0_; b
4	film thicknesses	Pd (d_M_)	ΔQ_te_ (C); Δφ_ms_(C)
5		Ta_2_O_5_ (d_1_)	ΔQ_te_(C); Q_te0_; C_0_; b
6		SiO_2_ (d_2_)	Q_te0_; C_0_; b
7	acceptor concentration	(N_A_)	a; φ_s0_; Q_ss_
8	channel length	(L)	b
9	channel width	(w)	
10	electron mobility in the channel	(µ_n_)	
11	relative dielectric permittivity	Ta_2_O_5_ (ε_1_)	a; b; C_0_
12		SiO_2_ (ε_2_)	
13		Si (ε_3_)	

**Table 3:** Average values of parameters for different *C*_0_ and (*w*/*L*) at *I_D_* is 0.1 mA and *V_D_* is 0.2 V.

Parameters →	*δC_0_*, %	*a*, *V*	δ*a*, %	*w*/*L* Is 0.003			*w*/*L* Is 0.006		
↓ *C_0_*, nF/cm^2^				*b*, mA/*V*^2^	δ*b*, %	Δ*V_G_*_0_, m*V*	*b*, mA/*V*^2^	δ*b*, %	Δ*V_G_*_0_, m*V*
30	2.1	1.37	3.1	1.62	7.3	6	3.24	7.0	3
40	3.6	1.02	4.6	2.16	8.8	4.6	4.32	8.5	2.3
50	5.5	0.82	6.5	2.70	10.4	3.7	5.4	10.1	1.8

**Table 4:** Average values of metrological characteristics for different *C*_0_ at δ*C_max_* being equal to 7%.

MC →	*V_G_*_0_, *V*	Δ*V_m_*, *V*	*k_C_*, 1/(vol.%)	*S_dmax_*, *V*/(vol.%)	δ*C_min_*, %	*C_th_*, ppm	*C*_1_, ppm	*C*_2_, ppm	*C_max_*, ppm
↓ *C*_0_, nF/cm^2^									
30	1.55	0.61	8	4.88	4.9	2.0	50	1410	8016
40	1.52	0.42		3.36	5.5	2.9	90	1210	7550
50	1.48	0.32		2.56	6.1	3.9	160	960	7210

## Data Availability

Data sharing not applicable.
